# Comparison of Efficacy of Dexamethasone and Methylprednisolone in Improving PaO2/FiO2 Ratio Among COVID-19 Patients

**DOI:** 10.7759/cureus.10918

**Published:** 2020-10-12

**Authors:** Muhammad A Rana, Mubashar Hashmi, Ahad Qayyum, Rizwan Pervaiz, Muhammad Saleem, Muhammad Faisal Munir, Muhammad Muneeb Ullah Saif

**Affiliations:** 1 Internal Medicine, Bahria International Hospital, Lahore, PAK; 2 Medical Education and Simulation, Bahria International Hospital, Lahore, PAK

**Keywords:** sars-cov-2, covid-19, inflammatory markers, p/f ratio, dexamethasone, methylprednisolone, ratio of partial pressure of arterial oxygen and fraction of inspired oxygen, oxygenation, covid induced ards, cytokines release syndrome

## Abstract

Introduction

Severe acute respiratory syndrome coronavirus 2 (SARS-CoV-2) is the reason for the global pandemic that started from Wuhan, China, in December 2019, known as coronavirus diseases 2019 (COVID-19). Acute respiratory distress syndrome happened in COVID-19 not just because of uncontrolled viral replication but also because of an uncontrolled immune reaction from the host. That’s why antiviral and anti-inflammatory treatments have become an increasing concern for clinicians.

Methods

A retrospective quasi-experimental study design was used to assess the effectiveness of methylprednisolone and dexamethasone in the improvement of PaO_2_/FiO_2_ (P/F) ratio in COVID-19 patients. We included 60 participants for this study by using a convenient sampling technique and divided them into two groups with 30 patients in each group. Group 1 was given dexamethasone 8 mg twice daily, and group 1 given methylprednisolone 40 mg twice daily for eight days. We recorded C-reactive protein (CRP), serum ferritin level, and P/F ratio before administration of both drugs and after administration of drugs for eight days. We used the paired t-test to assess the effect of both drugs on the P/F ratio of participants.

Results

The initial mean CRP in group 1 was 110.34, which reduced to 19.45 after administration of dexamethasone; similarly, the CRP in group 2 was 108.65, which reduced to 43.82 after administering methylprednisolone for eight days. In P/F ratio improvement, the calculated significance value for dexamethasone (p=0.000) was less than the table value at 0.05 in all sections, p-value for methylprednisolone (p=0.009) was also less than the table value at 0.05, which shows that both dexamethasone and methylprednisolone were effective in improving P/F ratio. Calculated p-value for dexamethasone (p=0.000) was lower than the calculated p-value for methylprednisolone (p=0.009), which shows that dexamethasone is more effective as compare to methylprednisolone.

Conclusions

Steroid therapy is effective in controlling inflammation markers, and especially dexamethasone is significantly effective in improving the P/F ratio in COVID-19 patients.

## Introduction

The coronavirus disease 2019 (COVID-19) pandemic caused by the severe acute respiratory syndrome coronavirus 2 (SARS-CoV-2) originated in Wuhan, China, in December 2019. Although most infected patients undergo an uneventful recovery, approximately 19% of patients experience severe pneumonia and 14% experience a progressive worsening to critical pneumonia [1].

Patients with severe COVID-19 quickly progressed to acute respiratory failure, pulmonary edema, and acute respiratory distress syndrome (ARDS) [2], which occurred not just because of an uncontrolled viral replication but also because of an uncontrolled immune reaction from the host. With the existence of uncontrolled viral replication, the presence of an increased number of damaged epithelial cells and cell debris activates a massive cytokine release, also known as a cytokine storm, with hyperinflammation and immune inhibition, which are characterized by decreased memory cluster of differentiation-4 þ T helper cells and an increased cluster of differentiation-8 cytotoxic activity [3].

As a result, antiviral and anti-inflammatory treatments have become an increasing concern for clinicians [4]. A randomized clinical study demonstrated that corticosteroid therapy could reduce inflammatory responses, treatment failure, and the time to clinical stability in community-acquired pneumonia without major adverse effects [5].

Recently, Villar et al. reported that early administration of dexamethasone shortened mechanical ventilation time and overall mortality for patients with moderate-to-severe ARDS [6]. Corticosteroid therapy was associated with improved clinical outcomes in severe COVID-19 patients in clinical practice. Zhou et al. reported that corticosteroid therapy improved the clinical symptoms and oxygenation of patients with COVID-19 [7].

Wang et al. also found that for patients with severe COVID-19, corticosteroid therapy reduced hospital length of stay and intensive care unit (ICU) stay [8]. Chinese experts considered it prudent to administer short courses of corticosteroids at low-to-moderate doses for critically ill patients with COVID-19 [9]. Wu et al. reported that treatment with methylprednisolone decreased the risk of death for individuals with COVID-19 with ARDS [10].

Objective

Using steroids in moderate-to-severe disease is a standard practice in the management of COVID-19, and we have been following the same. Some cases were treated with methylprednisolone, whereas others were treated with equivalent doses of dexamethasone. This has automatically created a patient pool consisting of two groups.

The objective of this study was to assess the effectiveness of dexamethasone and methylprednisolone in COVID-19 patients and to compare both drugs in regard to partial pressure arterial oxygen and fraction of inspired oxygen (PaO_2_/FiO_2_ [P/F]) ratio improvement. A significant amount of research has been published on the effectiveness of steroids in COVID-19 patients, but there is limited literature comparing the effectiveness of dexamethasone and methylprednisolone.

Hypothesis

We created two hypotheses: the H1 (alternate hypothesis) and the H0 (null hypothesis). The H1 hypothesis states that methylprednisolone and dexamethasone when given individually (either dexamethasone or methylprednisolone) can improve the P/F ratio in COVID-19 patients. The H0 hypothesis states that methylprednisolone and dexamethasone cannot improve the P/F ratio in COVID-19 patients.

## Materials and methods

We used a retrospective quasi-experimental study design to assess the effectiveness of methylprednisolone and dexamethasone in improving the P/F ratio in COVID-19 patients. We used a convenient sampling technique to select files (medical record) of 60 participants, all of whom had been admitted and treated in the high-dependency unit (HDU)/ICU and had been on bi-level positive airway pressure. The medical records of these 60 patients were divided into two groups: group 1, who had received dexamethasone, and Group 2, who had received methylprednisolone. Each group consisted of medical records of 30 patients. It needs to be reemphasized that these cases were already treated and had been concluded. They were treated by different treating teams. One treating group was pro-dexamethasone who used to give dexamethasone to their patients, whereas the other team had been favoring methylprednisolone and used it in equivalent doses to dexamethasone. Apart from the type of steroid used, all other management was same for both groups of patients including anticoagulation, and we would like to declare that both groups were on therapeutic anticoagulants. We had administered dexamethasone 8 mg twice daily to one group (labelled as group 1) participants and methylprednisolone 40 mg twice daily (almost equivalent dose to dexamethasone) to now labelled as group 2 participants. The duration of treatment was eight days. Laboratory specimens were being sent daily during the morning shifts to enable the treating clinicians to take decisions during their treatment periods. We chose two sets of laboratory values for this study, with one set that was taken on the first day of hospitalization and the second set from eighth day of administration of drugs, from their medical records. Similarly, for the P/F ratio comparison, we took the PaO2 values from their daily morning arterial blood gas reports, two sets again. For data analysis, we described demographic data in descriptive form (frequencies and percentages); laboratory values were analyzed in the form of mean, and comparison between dexamethasone and methylprednisolone effectiveness of the P/F ratio was analyzed using a paired t-test.

## Results

Our results are presented in three parts. part I includes demographic characteristics of participants, part II includes outcomes of laboratory value changes in response to dexamethasone and methylprednisolone, and part III includes a comparison of outcomes in response to both drugs.

Part I

**Figure 1:**
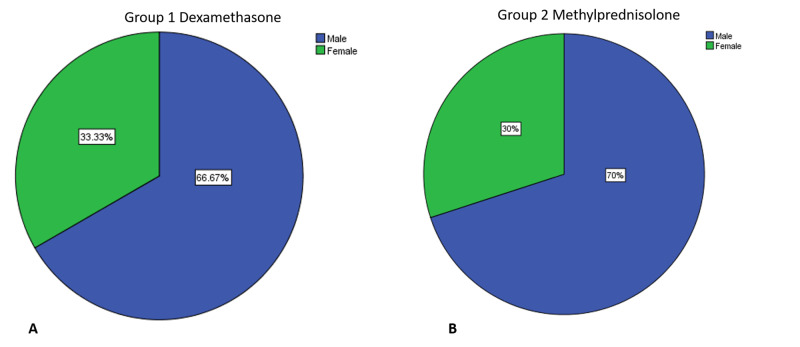
Sex distribution for group 1 (A) and group 2 (B)

Medical records of 60 patients were selected for this study, with 30 participants in each group: group 1, which had received dexamethasone for eight days, and group 2, which had received methylprednisolone for eight days. Both groups were on therapeutic anticoagulants. In group 1, 33.33% were women and 66.67% were men; similarly, in group 2, 30% were women and 70% were men (Figure 1). The mean age of group 1 was 53.8 years, and the mean age of group 2 was 53.9 years.

Part II

In this section, we discuss the changes that occurred in laboratory values of patients before and after administration of dexamethasone and methylprednisolone and how effectively dexamethasone and methylprednisolone worked. In Table 1, the C-reactive protein (CRP) difference is shown. The initial mean CRP in group 1 was 110.34 mg/L, which decreased to 19.45 mg/L after administration of dexamethasone and was considered a good reduction in CRP value. Similarly, the CRP in group 2 was 108.65 mg/L, which decreased to 43.82 after the administration of methylprednisolone for eight days. In Table 2, serum ferritin levels are shown. The initial mean serum ferritin level in group 1 was 763 ng/mL, which decreased to 494.30 ng/mL after administration of dexamethasone for eight days. Similarly, the serum ferritin level in group 2 was 631.53 ng/mL, which decreased to 493.07 ng/mL after administration of methylprednisolone for eight days.

**Table 1:** Comparison of CRP before and after the use of methylprednisolone and dexamethasone. CRP, C-reactive protein

	Mean	N
CRP before dexamethasone	110.34 mg/L	30
CRP after dexamethasone	19.45 mg/L	30
CRP before methylprednisolone	108.65 mg/L	30
CRP after methylprednisolone	43.82 mg/L	30

**Table 2:** Comparative values of ferritin levels before and after the use of methylprednisolone and dexamethasone.

	Mean	N
Ferritin pre-dexamethasone	763.00 ng/mL	30
Ferritin post-dexamethasone	494.30 ng/mL	30
Ferritin pre-methylprednisolone	631.53 ng/mL	30
Ferritin post-methylprednisolone	493.07 ng/mL	30

The focus of this study was the evaluation of the P/F ratio in both groups after the administration of methylprednisolone and dexamethasone. As shown in Table 3, the P/F ratio of group 1 at the initial stage was 118.20, which improved to 170.41 after the administration of dexamethasone for eight days. Both values were in the range of moderate respiratory failure, but somehow the P/F ratio improved. Similarly, in group 2, the P/F ratio at the initial stage before methylprednisolone therapy was 105.66, which improved to 136.25 after the administration of methylprednisolone.

**Table 3:** P/F ratio before and after the use of methylprednisolone and dexamethasone. P/F, PaO_2_/FiO_2_

	Mean	N
P/F ratio pre-dexamethasone	118.20	30
P/F ratio post-dexamethasone	170.41	30
P/F ratio pre-methylprednisolone	105.66	30
P/F ratio post-methylprednisolone	136.25	30

Part III

This section consisted of outcomes comparison after administering methylprednisolone and dexamethasone. As mentioned previously, the main focus of this study was to assess the effectiveness of dexamethasone and methylprednisolone on the P/F ratio. To compare the pre- and post-P/F ratio, we applied a paired sample t-test to check the effectiveness of both drugs. Findings revealed that the mean P/F ratio after dexamethasone (170.41) was significantly better than the mean P/F ratio before dexamethasone (118.20; p=0.000). Similarly, the P/F ratio after methylprednisolone (136.25) had improved than the P/F ratio before administration of methylprednisolone (105.66) for eight days (p=0.009; Table 4). As these p-values indicate, dexamethasone improved the P/F ratio significantly more than methylprednisolone.

**Table 4:** Tabulated description of improvement in P/F ratio before and after the use of methylprednisolone and dexamethasone. Values derived using paired sample statistics P/F, PaO_2_/FiO_2_

		Mean	N	Standard Deviation
Pair 1	P/F ratio before dexamethasone	118.20	30	59.22
	P/F ratio after dexamethasone	170.41	30	76.04
Pair 2	P/F ratio before methylprednisolone	105.66	30	43.86
	P/F ratio after methylprednisolone	136.25	30	73.40

The H1 alternate hypothesis (methylprednisolone and dexamethasone are effective in improving P/F ratio in COVID-19 patients) can be accepted, and the H0 null hypothesis (methylprednisolone and dexamethasone could not improve the P/F ratio in COVID-19 patients) can be rejected on the basis of the paired t-test (Table 5).

**Table 5:** Paired sample statistics, with p-values indicating that dexamethasone improved the P/F ratio significantly more than methylprednisolone. Values derived using paired sample test Df, degrees of freedom; P/F, PaO_2_/FiO_2_

		Paired Differences					t	df	Significance (two-tailed)
		Mean	Standard Deviation	Standard Error Mean	95% Confidence Interval of the Difference				
					Lower	Upper			
Pair 1	Pre-P/F ratio dexamethasone	-52.20	50.07	9.14	-70.90	-33.509	-5.71	29	0.000
	Post-P/F ratio dexamethasone								
Pair 2	Pre-P/F ratio methylprednisolone	-30.59	60.26	11.00	-53.10	-8.091	-2.78	29	0.009
	Post-P/F ratio methylprednisolone								

## Discussion

The mean age of patients in this study was 53.3 years, and most patients (66.67%) were men and 33.33% were women. A similar study conducted by Spagnuolo et al. showed that the median age of participants was 63.5 years (range: 53.5 to 74 years), and 34% of patients were older than 70 years [11].

We found that corticosteroids reduced CRP levels. A similar study conducted in 2005 indicated that the withdrawal of inhaled corticosteroids increased serum CRP levels. The reintroduction of the inhaled steroids suppressed the CRP levels. Hence, corticosteroids can reduce serum CRP and other circulating inflammatory cytokine levels in some acute inflammatory states [12].

In this study, the initial mean serum ferritin levels in both drug groups were reduced after eight days. A study conducted on *Mycoplasma pneumoniae* in November 2015 elaborated that ferritin levels may be useful as indicators of the severity of pneumonia for initiation of corticosteroid therapy [13].

The findings of this study revealed that both dexamethasone and methylprednisolone were effective in improving the P/F ratio. A study conducted by Spagnuolo et al. assessed the effectiveness of steroids in COVID-19 patients, where differences between patients taking steroids and patients not taking steroids were observed [11]. A remarkable improvement in the P/F ratio was observed. The proportion of patients with a baseline PaO_2_/FiO_2_ ≤ 200 mmHg was 45.8% for patients taking steroids compared to 34.4% in patients not taking steroids (p=0.023). The proportion of patients with a baseline PaO_2_/FiO_2_ ≤ 100 mmHg was 16.9% for patients taking steroids compared to 12.7% who were not taking steroids (p=0.027). Steroid use also decreased the length of hospitalization (20 vs. 14 days; p<0.001) [11].

There were certain limitations of the present study. We did not include a control group, did not assess any underlying comorbidities, and did not compare the effectiveness of steroid therapy in regard to age, sex, and severity of illness. Randomized controlled clinical trials are required to confirm the effectiveness and safety of corticosteroid therapy and to further study the long-term outcomes after discharge with a large sample size for better generalization of findings.

## Conclusions

Steroids have long been known to reduce inflammation and suppress immune reactions. These actions make steroids an effective tool in the treatment of COVID-19, and they have become a standard of treatment all over the world. Dexamethasone is more effective in improving the P/F ratio in COVID-19 patients compared to methylprednisolone. Improvement in the P/F ratio is inevitably associated with reduction in oxygen requirements, and this can lead to reduced length of stay in HDU or ICU, which favors outcomes as it has a profound effect on overall hospital length of stay. Moreover, as dexamethasone is a much cheaper drug and is widely available, physicians should consider the use of dexamethasone use in appropriate patients with COVID-19.
